# Arterio-Ureteral Fistula: A Rare Elusive Cause of Significant Hematuria

**DOI:** 10.7759/cureus.39989

**Published:** 2023-06-05

**Authors:** Muhammad Waqar, Suresh-Jay Mathias, Vinodh Murali, Helen Thursby, Christopher Luscombe, Ronald James, David Mak, Aniruddha Chakravarti, Christopher Day, Jules Dyer

**Affiliations:** 1 Urology, The Royal Wolverhampton NHS Trust, Wolverhampton, GBR; 2 Urology, University Hospitals of North Midlands NHS Trust, Stoke-on-Trent, GBR; 3 Urological Surgery, The Royal Wolverhampton NHS Trust, Wolverhampton, GBR; 4 Radiology, University Hospitals of North Midlands NHS Trust, Stoke-on-Trent, GBR; 5 Interventional Radiology, The Royal Wolverhampton NHS Trust, Wolverhampton, GBR

**Keywords:** abnormal ureteric connection, arterial bleed, urological emergency, arterioureteral fistulation, haematuria

## Abstract

Arterioureteral fistula (AUF) is a direct communication between the ureter and an artery and is a rare cause of catastrophic, life-threatening haematuria. Fistulation may occur between the ureter and the abdominal aorta, common iliac, external and internal iliac, and inferior mesenteric arteries, and is typically observed in patients with a prior history of pelvic radiotherapy, oncological pelvic surgeries, aortoiliac vascular procedures, and pelvic exenteration. There is also an increased frequency of cases amongst patients who have undergone urological diversion surgeries and in those with chronic indwelling ureteric stents requiring repeated exchange. As AUF is so rarely encountered in clinical practice, the urologist may fail to appreciate its presence until late in the patient’s presentation; such diagnostic delay is associated with high mortality and thus rapid clinical suspicion and investigative action are necessary. There are sporadic cases of this rare entity mentioned in literature. In this report, we present two cases as well as a review of the literature.

A 73-year-old female presented with repeated episodic haematuria for a week in whom the cause of symptoms remained persistently elusive despite repeated imaging and operative approaches. An eventual diagnosis of a secondary right internal iliac-ureteral fistula was ascertained on a subsequent digital subtraction angiography of the renal tract. The fistula was embolised using an endovascular approach. The patient remained stable post emobilisation and was successfully discharged shortly after the procedure. In the second case, a 51-year-old female, presented with hematuria from her ileal conduit for a few days. Initially, the cause of symptoms was thought to be due to ureteric stents. During a change in her stents, brisk bleeding led to further investigation including an iliac angiogram confirming bleeding from the left common iliac artery. She had a covered common iliac artery stent, which successfully controlled her bleeding

This report emphasizes the diagnostic difficulty of AUF, outlines the management principles of this rare disease, and aims to increase awareness of this rare yet potentially lethal phenomenon among practitioners of urology and interventional radiology

## Introduction

Arterioureteral fistula (AUF) is a seldom encountered but important cause of episodic large-volume haematuria that can lead to haemorrhagic shock and fatal exsanguination if not diagnosed and managed promptly. Fistulous communication occurs between the ureter and the following arterial structures: the abdominal aorta, common, external and internal iliacs, and the inferior mesenteric [1]. Arterial haemorrhage into the urinary tract accounts for the clinical presentations of haematuria and circulatory collapse. Because of its rare occurrence, awareness of AUF is lacking and its diagnosis can be challenging even to senior urological surgeons; mortality and morbidity are thus understandably high [2]. Discerning AUF from other common causes of haematuria requires a high level of suspicion and inclusion of AUF in differential diagnostic thinking enables both early detection and favourable prognosis. The purpose of this case series was to report and analyze two cases managed in our hospital which were compared to the results of an exhaustive literature review of AUF. We also describe risk factors, diagnosis, treatment, and the urologist’s point of view on AUF management

## Case presentation

Case 1

A 73-year-old female presented to our tertiary hospital with urethral haematuria and haematuria in both nephrostomies. She had a past medical history of squamous cell carcinoma of the cervix treated with pelvic radiotherapy in the 1970s, chronic kidney disease stage 3, peripheral vascular disease, and abdominal aortic aneurysm. She had been managed for several years with bilateral percutaneous nephrostomy tubes followed by bilateral ureteric stents requiring frequent exchanges. The bilateral ureteric obstruction was thought to be radiotherapy-associated long-segment bilateral distal ureteric strictures based on her retrograde studies. Her surgical history included one laparotomy with left hemicolectomy and formation of an end colostomy in 2005 performed for previous bowel obstruction, a second laparotomy for radiation enteritis with bypass of the ileum in 2013, and insertion of a dynamic hip screw for fractured neck of femur in 2016. 

**Figure 1:**
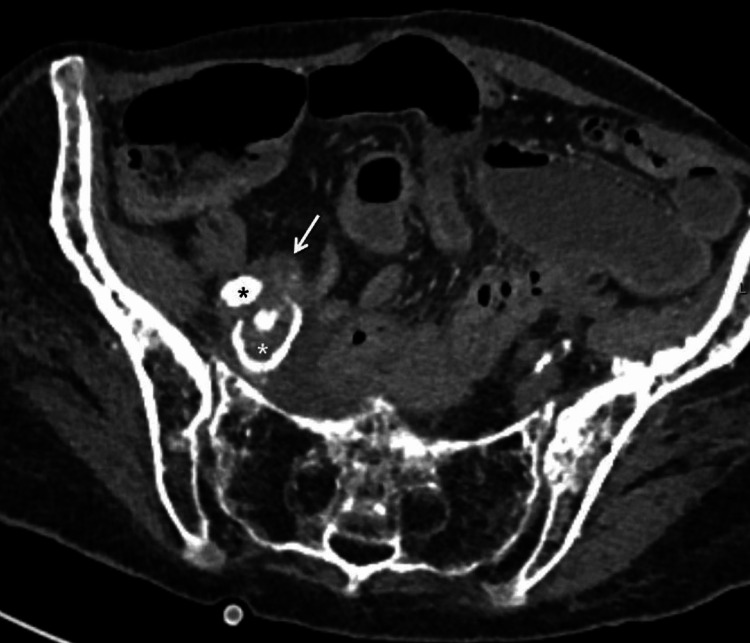
Axial CT angiogram. The ureter (white arrow) is inseparable from the external iliac artery (black asterisk) and ectatic internal iliac artery (white asterisk). No active extravasation of contrast seen at the time of the scan.

**Figure 2:**
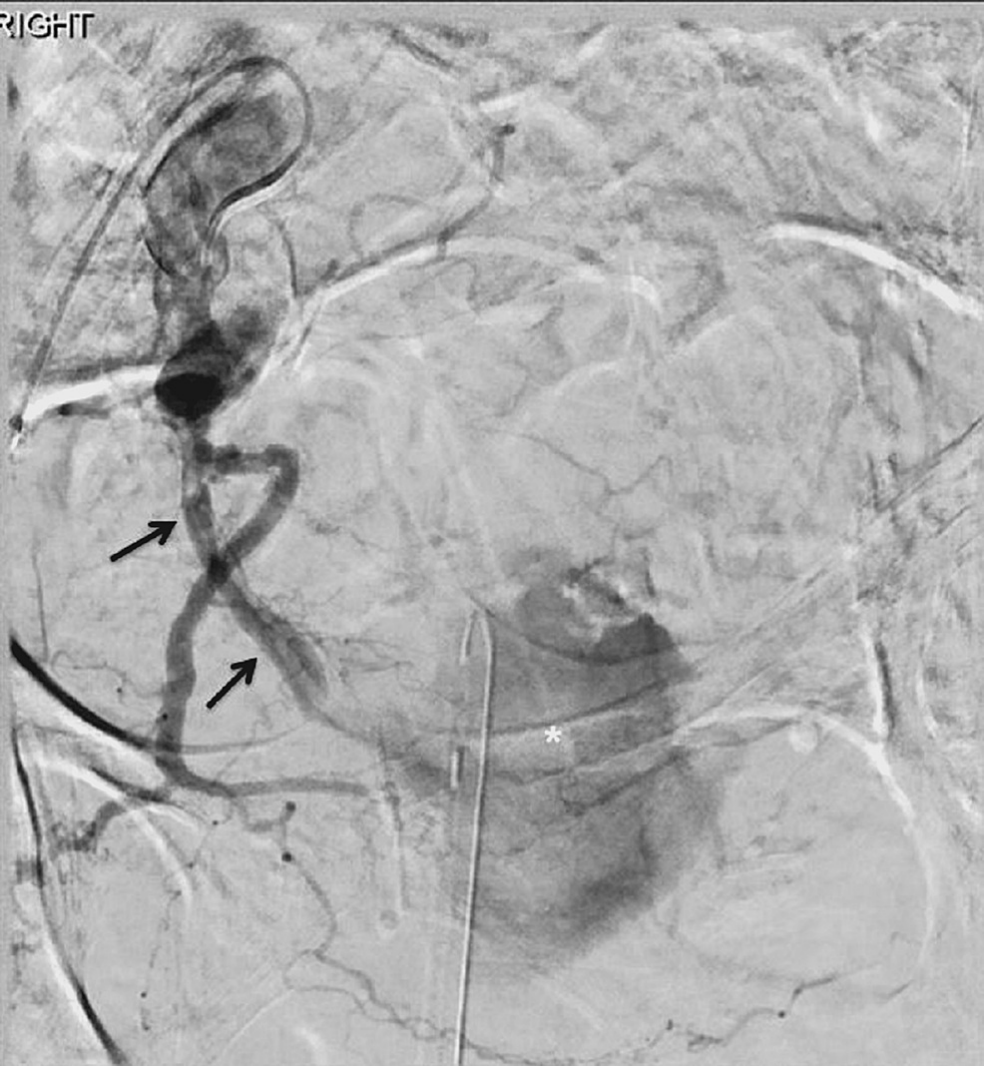
Cather angiogram from the internal iliac artery origin demonstrates active bleeding from the right internal iliac artery into the distal ureter (black arrows). A linear filling defect is seen within the ureter consistent with non-occlusive thrombus. Contrast is seen filling the urinary bladder (white asterisk).

**Figure 3:**
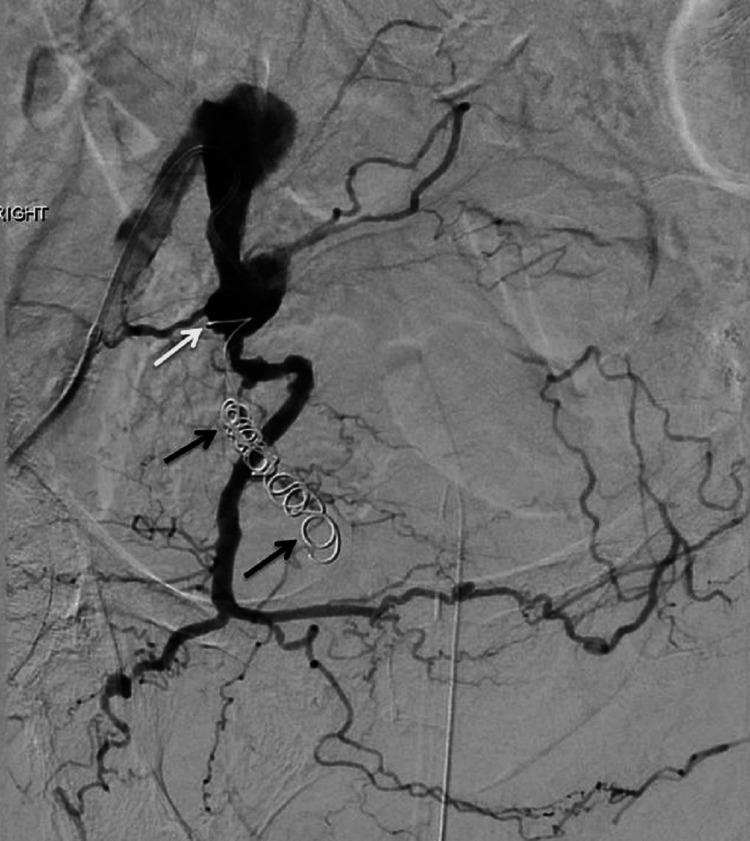
Post embolization. Embolization coils have been placed in the right distal ureter (black arrows) with the proximal end of the coils in the internal iliac artery across the fistula (white arrow). Bleeding has been successfully treated with no contrast entering the ureter or bladder.

**Figure 4:**
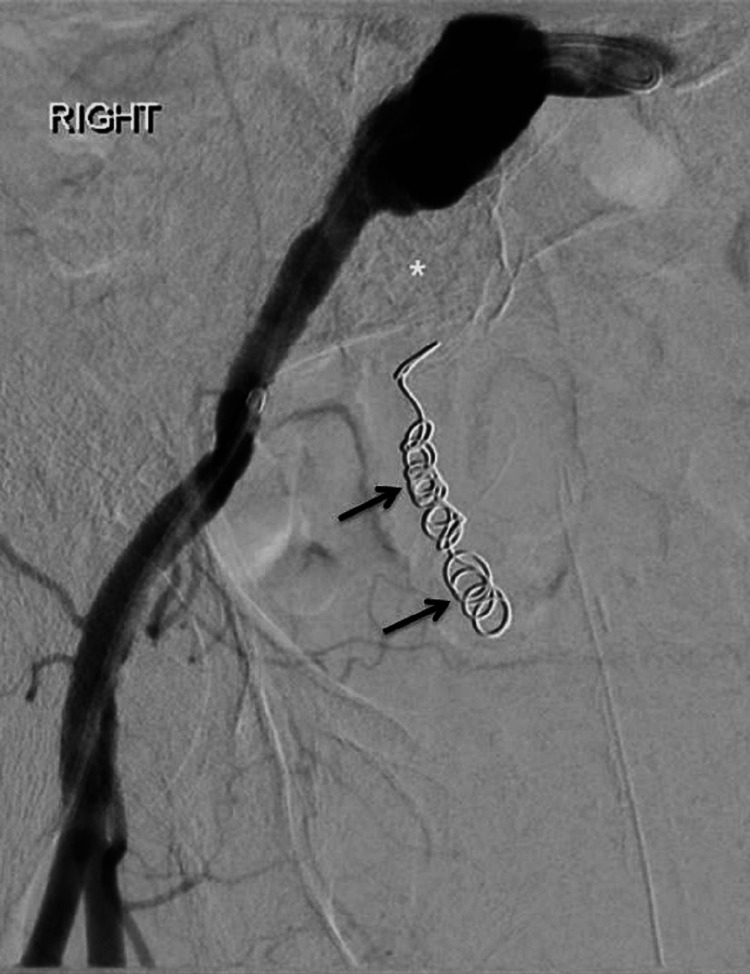
Day 8 post embolization. The internal iliac artery (white asterisk) and its branches are occluded with no contrast entering the distal right ureter that was preciously embolised with coils (black arrows).

The patient presented to the emergency department with severe haematuria and was hypotensive and tachycardic. Intravenous access was obtained and she was fluid resuscitated and a three-way urethral catheter was inserted and drained dark haematuria. Arterial blood gas sampling was performed along with the submission of routine venous blood samples for urea and electrolytes, full blood count, clotting profile and group, and save and crossmatch. Arterial blood gas analysis showed a haemoglobin level of 87 grams per deciliter (g/dL), an acute drop from the patient’s normal levels. Laboratory blood results showed an international normalised ratio of 1, activated partial thromboplastin time of 0.75 seconds, normal platelets, and stable creatinine levels. She was transfused with two units and post transfusion, haemoglobin levels recovered to 101 g/dL. The patient was then transferred to the care of the urology team following stabilisation. A decision to perform emergency computerized tomography angiography (CTA) of the renal and abdominal vessels was undertaken. Figures 1-2 show the diagnostic imaging. AUF can be seen in Figure 2. Figures 3-4 show post-treatment scans after successful embolization.

Case 2

A 51-year-old female with a past medical history of locally advanced cervical cancer treated with radiotherapy and pelvic exenteration in 2012, had ileal conduit urinary diversion in 2013 and bowel resection and anastomosis in 2014 for bowel obstruction. She had revision of the ileal conduit with further bowel resection in 2017. She developed bilateral utero-ileal anastomotic strictures, which were managed with long-term bilateral ureteric stents requiring frequent exchanges.

**Figure 5:**
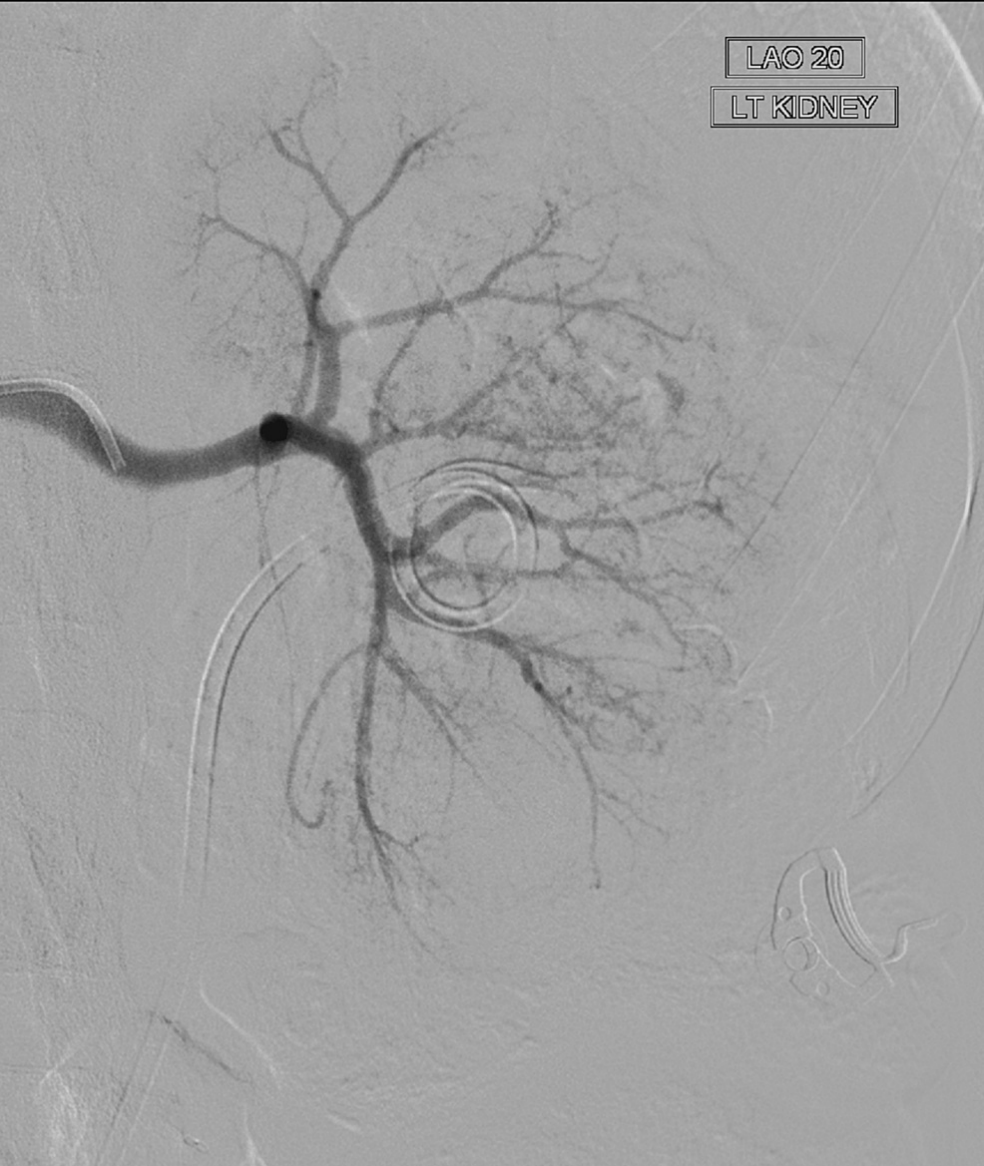
Catheter right renal angiogram following torrential bleed and pain in left flank. No active bleed, no pseudoaneurysm, and no spasm.

**Figure 6:**
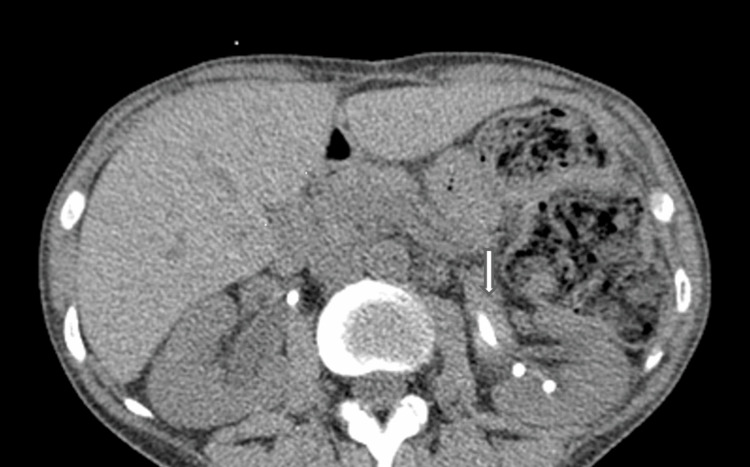
Plain CT showing increased density within a dilated left renal tract (white arrow) indicating distension with blood/acute thrombus.

**Figure 7:**
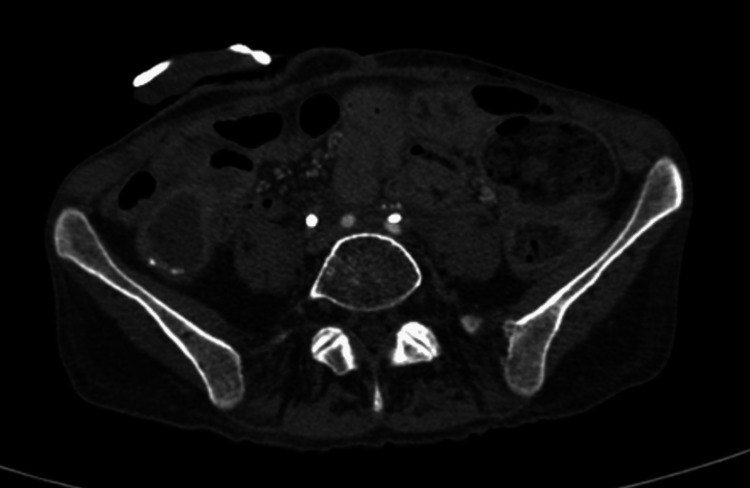
Contrast-enhanced CT (arterial phase). There is no plane between the ureteric stent and the left iliac artery.

The patient presented to the emergency department with haematuria in the stoma requiring blood transfusion. After initial resuscitation, she was transferred to the urology department. Initially, the stent was thought to be the culprit. However, CTA surprisingly showed the whole left collecting system full of clots but did not show any false aneurysm or active bleeding point (Figures 5-7).

**Figure 8:**
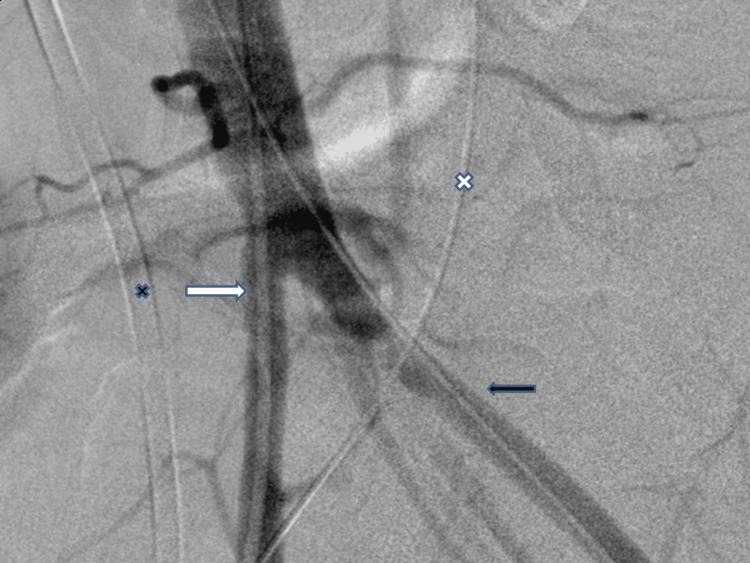
Catheter iliac angiogram with a diagnostic catheter in the right iliac artery (white arrow), a wire in the left (black arrow), a plastic stent in the right ureter (black cross), and a wire in the left (white cross). Note the stenosis in the left iliac artery.

**Figure 9:**
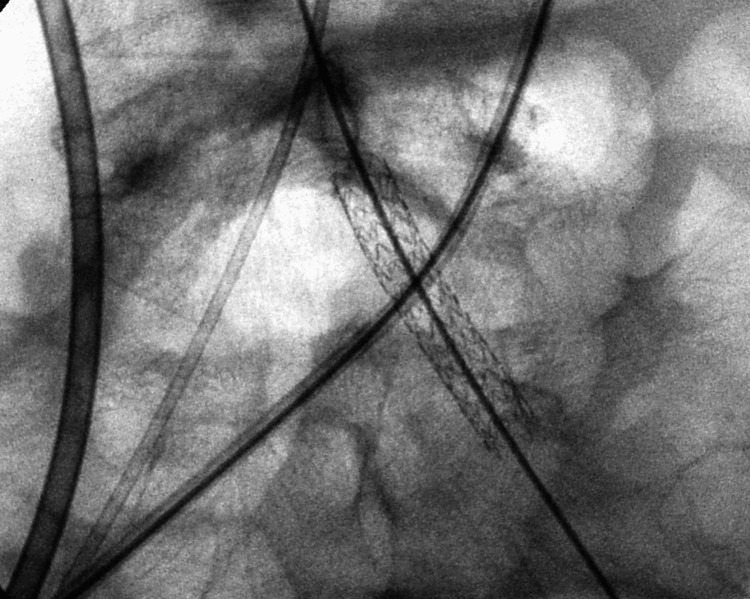
Balloon-mounted covered stent deployed.

The interventional radiology team was requested to change her stent. During the procedure, brisk bleeding was noticed when half of the stent was pulled out and it stopped when replaced back. A more specific iliac angiogram was performed while the left ureteric stent was withdrawn which confirmed bleeding from the left common iliac artery. Each bleed was followed by acute flank pain. Ultimately, the diagnosis of left uretero-iliac artery fistula was confirmed and the patient had covered a common iliac artery stent which successfully controlled her bleeding (Figures 8-9).

Both cases were successfully managed with assistance from interventional radiology. The first case had successful embolization while the second one had successful arterial stent.

## Discussion

The presenting symptom common to all patients with AUF is brisk large volume haematuria which could be intermittent, the timing of which is variable. Some patients elicit sporadic episodes of haematuria with subsequent significant drops in haemoglobin necessitating transfusion followed by quiescent periods; others may present haemodynamically compromised with massive haematuria and no prior clinical signs. The latter is more unusual. Pain is a variable feature and occurs due to clot organisation within the renal pelvis and ureter following bleeding episodes. Thus, intermittent haematuria with painful bleed-free intervals comprises a typical AUF presentation. Haematuria intermittency can be attributed to clot formation and occlusion of the fistulous communication between the ureter and artery [3]. Breakdown of the clot leads to recurrence of bleeding through the urinary outflow tract.

Aetiology

AUFs can be classified as primary, secondary/iatrogenic or pregnancy related. Pregnancy-related AUF is vanishingly rare.

Primary AUFs are less common and are almost always seen as a consequence of aortoiliac aneurysmal disease in which there is direct erosion of the arterial aneurysm or pseudoaneurysm into the ureter [4-6] although congenital iliac arteriovenous malformation has been reported as a cause of the primary AUF variant [7]. Aneurysmal rupture in this setting leads to direct haemorrhage into the ureter and urinary outflow tract with resultant gross haematuria. 

The vast majority of AUFs encountered in clinical practice are of the secondary subtype. These are iatrogenic fistulae and develop as sequelae of combined pelvic radiotherapy and surgery for urological/colorectal or gynaecological malignancy. Surgical intervention for uterine cancer and transitional carcinoma of the bladder incur a particularly higher risk of secondary fistulation. Secondary AUF has also been well documented as a complication in patients with chronic urinary outflow obstruction with hydronephrosis requiring repeated exchanges of ureteral stents and in those requiring repeated stenting post ureteral dilatation for ureteral strictures. Prolonged use of rigid ureteral stents and catheters (eg., metallic stents) are associated with higher incidences of secondary AUF development [8]. Other documented causes of iatrogenic-induced fistulae include the presence of a ureteral stump post nephrectomy, vascular reconstructive surgery with the use of grafts following aortic and iliac aneurysm repair, and renal transplantation surgery. In the latter, a secondary AUF between the renal graft aneurysm and the recipient ureter was observed [9]. Urinary leak post ureterolithotomy has also been seen. 

Pregnancy-related AUF is a rare variant of the primary fistula subgroup, with only three cases documented within the literature. These fistulae were all identified in post-mortem studies with the pathogenic mechanism involving chronic ureteropelvic obstruction secondary to severe pyelonephritis. Fortunately, this aetiology seems to have become redundant now that liberal antibiotic use is commonplace in urinary tract infections during pregnancy [10]. 

Pathophysiology

The pathophysiological mechanism underlying the formation of AUF is not fully understood, but both inflammatory and mechanical components have been postulated. Peri-ureteric inflammation caused through surgical manipulation of the ureter, external beam radiotherapy, or urinary leakage (e.g. post ureterolithotomy) may cause a relative fixation of the ureter to the artery (or vascular graft in patients having undergone vascular reconstruction). Additionally, radiotherapy and pelvic surgery may directly damage arterial vasa vasorum and the ureteral blood supply, decreasing the threshold for arterial necrosis and inducing ureteral devascularisation respectively. Inflammatory-mediated relative fixation of devascularised ureter to artery then allows for mechanical pulsatile micro-trauma to be transmitted to the ureter from the artery. Synthetic vascular grafts and indwelling rigid ureteral stents increase the risk of pulsatile pressure injury. This repeated pulsatile ureteral microtrauma progresses to pressure necrosis with subsequent fistulous communication [11].

## Conclusions

AUF is a rare but lethal condition. Its occurrence is strongly linked with risk factors such as abdominal/pelvic surgical intervention, abdominal radiation, and urethral manipulation, especially long-term urethral stent. The pathophysiology is unknown but the inflammatory response generated by interventional procedures creates a connection between the ureter and artery. Due to its rare occurrence, its diagnosis is challenging but anyone presenting with brisk haematuria should be investigated for AUF with proper radiological investigation, ideally an angiogram. and treated either with arterial stent or embolization depending on the aetiology. Untreated cases could have catastrophic outcomes.

## References

[1] Mujo T, Priddy E, Harris JJ, Poulos E, Samman M (2016). Unique presentation of hematuria in a patient with arterioureteral fistula. Case Rep Radiol.

[2] Turo R, Hadome E, Somov P, Hamid B, Gulur DM, Pettersson BA, Awsare NS (2018). Uretero-arterial fistula - not so rare?. Curr Urol.

[3] Chantada Abal V, Gómez Veiga F, Sousa Escandon A, González Martín M (1993). Common iliac artery-ureteral fistula: a case report and literature review. Arch Esp Urol.

[4] Mahoney PF, Stephen JG (1987). External iliac artery-ureteric fistula. Br J Urol.

[5] Grime PD, Wilmshurst CC, Clyne CA (1989). Spontaneous iliac artery aneurysm—ureteric fistula. Eur J Vasc Surg.

[6] Batter SJ, McGovern FJ, Cambria RP (1996). Ureteroarterial fistula: case report and review of the literature. Urology.

[7] Sharma SK, Goswami AK, Sharma GP, Malakondiah GC, Khanna SK (1988). Congenital iliac arteriovenous malformation: a cause of massive hematuria and ureteral obstruction. J Urol.

[8] Adams PS Jr (1984). Iliac artery-ureteral fistula developing after dilatation and stent placement. Radiology.

[9] List A, Collins J, MacCormick M ( 1990). Massive hemorrhage from an arterioureteral fistula associated with chronic renal transplant failure. J Urol.

[10] Bergqvist D, Pärsson H, Sherif A (2001). Arterio-ureteral fistula--a systematic review. Eur J Vasc Endovasc Surg.

[11] Escobar PF, Howard JL, Kelly J, Roland PY, Grendys EC, Dosoretz DE, Orr JW Jr (2008). Ureteroarterial fistulas after radical pelvic surgery: pathogenesis, diagnosis, and therapeutic modalities. Int J Gynecol Cancer.

